# NEDD8-conjugating enzyme E2s: critical targets for cancer therapy

**DOI:** 10.1038/s41420-023-01337-w

**Published:** 2023-01-23

**Authors:** Lisha Zhou, Xiongzhi Lin, Jin Zhu, Luyi Zhang, Siyuan Chen, Hui Yang, Lijun Jia, Baofu Chen

**Affiliations:** 1 Taizhou Central Hospital (Taizhou University Hospital), Taizhou University, Taizhou, Zhejiang China; 2 Graduate School of Medicine, Hebei North University, Zhangjiakou, Hebei China; 3 Department of Surgical Oncology, Jiangxi Cancer Hospital, Nanchang, Jiangxi China; 4 Department of Neurosurgery, Huashan Hospital, Fudan University, Shanghai, China; 5 Cancer Institute of Traditional Chinese Medicine, Longhua Hospital, Shanghai University of Traditional Chinese Medicine, Shanghai, China

**Keywords:** Oncogenes, Tumour immunology

## Abstract

NEDD8-conjugating enzymes, E2s, include the well-studied ubiquitin-conjugating enzyme E2 M (UBE2M) and the poorly characterized ubiquitin-conjugating enzyme E2 F (UBE2F). UBE2M and UBE2F have distinct and prominent roles in catalyzing the neddylation of Cullin or non-Cullin substrates. These enzymes are overexpressed in various malignancies, conferring a worse overall survival. Targeting UBE2M to influence tumor growth by either modulating several biological responses of tumor cells (such as DNA-damage response, apoptosis, or senescence) or regulating the anti-tumor immunity holds strong therapeutic potential. Multiple inhibitors that target the interaction between UBE2M and defective cullin neddylation protein 1 (DCN1), a co-E3 for neddylation, exhibit promising anti-tumor effects. By contrast, the potential benefits of targeting UBE2F are still to be explored. It is currently reported to inhibit apoptosis and then induce cell growth; hence, targeting UBE2F serves as an effective chemo-/radiosensitizing strategy by triggering apoptosis. This review highlights the most recent advances in the roles of UBE2M and UBE2F in tumor progression, indicating these E2s as two promising anti-tumor targets.

## Facts

- More research is being devoted to finding specific inhibitors against neddylation E2s to address the limitations of neddylation E1 inhibitor MLN4924.
- UBE2M and UBE2F are overexpressed in various malignancies, conferring a worse overall survival.
- Targeting UBE2M to influence tumor growth by either modulating several biological responses of tumor cells (such as DNA-damage response, apoptosis, or senescence) or regulating the anti-tumor immunity holds strong therapeutic potential.
- A few inhibitors targeting UBE2M-DCN1 interaction have been identified.
- UBE2F appears to be promising as both an anti-tumor target and a chemo-/radiosensitizing target.

## Open questions

- The induction of drug-resistant mutations in UBA3 inhibits the formation of the MLN4924-NEDD8 adduct, and hence there is a need to explore possible alternative targets against the neddylation pathway.
- How do the two E2s regulate anti-tumor immunity? In-depth elucidation of the underlying mechanisms may provide vital cues for targeting UBE2M and UBE2F.
- Deciphering the cellular potency of these UBE2M-DCN1 inhibitors compared to MLN4924?
- Whether these UBE2M-DCN1 inhibitors have any biological function?
- Whether to promote clinical trials of these E2s inhibitors alone or in combination?

## Introduction

**Fig. 1:**
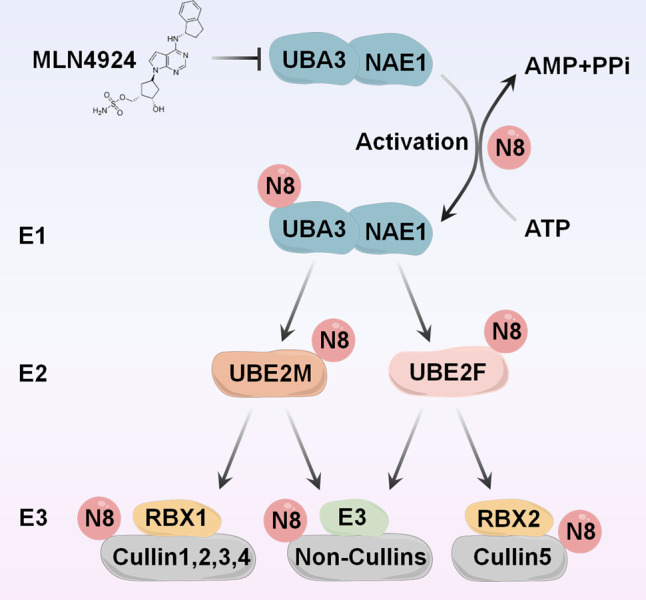
The process of protein neddylation. Neddylation is a process that conjugates NEDD8 to cullins or non-cullin substrates via a three-step reaction, catalyzed by NEDD8-activating enzyme E1 (a heterodimer of NAE1 and UBA3), NEDD8-conjugating enzyme E2 (UBE2M or UBE2F) and substrate-specific NEDD8-E3 ligases (e.g., RBX1 and RBX2). MLN4924: an inhibitor of UBA3; N8: neuronal precursor cell-expressed developmentally downregulated protein 8.

Protein neddylation involves the conjugation of a ubiquitin-like molecule known as neuronal precursor cell-expressed developmentally down-regulated protein 8 (NEDD8) to the lysine residue of targeted substrate proteins [1, 2]. Like ubiquitin, NEDD8 binds to substrates by forming an isopeptide chain between its C-terminal glycine residue (Gly76) and a lysine residue on targeted proteins. In the initial step of the reaction, NEDD8 is produced as a precursor containing five additional residues downstream from Gly76 cleaved by the C-terminal hydrolases [3, 4]. The next step involves activation of the mature NEDD8 in an ATP-dependent manner by the NEDD8-activating enzyme (NAE) E1, a heterodimer composed of NAE1 and ubiquitin-like modifier activating enzyme 3 (UBA3) [5, 6]. After that, a trans-thiolation process occurs during which the NEDD8-loaded NAE is transferred to either UBE2M, also known as UBC12, or UBE2F (two NEDD8-conjugating enzyme E2s) [7–9]. Finally, covalent attachment drives a substrate-specific E3 ligase, such as defective in cullin neddylation 1 (DCN1) and RING-box protein 1/2 (RBX1/2), to transfer NEDD8 from the charged E2 to a lysine residue in its target protein (Fig. 1) [10–14]. The best characterized principal substrates of neddylation are the cullin family members (CUL1, 2, 3, 4A, 4B, and 5), which function as core components of Cullin-RING E3 ubiquitin ligases (CRLs) [4]. The activation of CRLs requires NEDD8 to attach to a C-terminal lysine residue of cullins, inducing structural changes in the CRLs complex and leading to open conformation to facilitate substrate ubiquitylation [15–19]. As the most prominent family of E3 ubiquitin ligases, CRLs noticeably regulate various essential biological functions, including tumorigenesis, accomplished by enhancing ubiquitylation and consequent degradation of a wide range of critical modulatory proteins [4, 20]. Besides cullins, several non-cullin proteins have been identified as the substrates of neddylation, such as p53, MDM2, and EGFR [21–24]. However, regulatory mechanisms and physiological functions of non-cullin substrates still await experimental validation.

In multiple cancers, overactivation of the neddylation pathway leads to elevated global neddylation of substrates, such as cullins, and consequent accumulation of tumor suppressors, thereby promoting tumorigenesis and development [25–28]. Targeting the overactivated protein neddylation pathway has been proven to be an effective anti-tumor strategy. MLN4924, commonly referred to as pevonedistat, is an effective and highly selective small-molecule inhibitor of NAE adopted to inhibit protein neddylation via inactivation of the initial stage of the neddylation cascade [29]. When bound to the active site of UBA3, MLN4924 forms a stable covalent adduct with NEDD8 to block further enzymatic processes [30]. It achieves potent anti-tumor effects by inducing cell cycle arrest, apoptosis, or senescence of tumor cells, or affecting the functions of multiple components of the tumor microenvironment [28, 29, 31–37]. MLN4924 has been evaluated in Phase I/II clinical trials for treating various solid tumors and hematologic cancers [38–44]. Nevertheless, the induction of drug-resistant mutations in UBA3 inhibits the formation of the MLN4924-NEDD8 adduct, necessitating the need to explore alternative targets against the neddylation pathway [45, 46]. To address the limitations of MLN4924, specific inhibitors against neddylation E2s are being investigated [47, 48]. This review summarizes the latest progress on validating the neddylation of these enzymes as promising anti-tumor targets.

## Biological characteristics and correlation of UBE2M and UBE2F

UBE2M and UBE2F bind to the ubiquitin-fold domain and the UBA3 hydrophobic groove of E1 via the core domain and the N-terminal motif, respectively [9]. Acetylation of the N-terminal methionine occurs in both E2s, which facilitates their binding to the PONY domain pocket of neddylation E3 DCN-like (DCNL), thereby increasing the efficiency of the cullin neddylation process [49–51]. In addition to these similarities, protein structure assessment of both UBE2M and UBE2F reveals unique characteristics. UBE2F is very specific to the neddylation of RBX2-related CUL5, while UBE2M can pair with RBX1 to modulate the neddylation of CUL1, 2, 3, 4A, and 4B [50]. It is noteworthy that glycyl-tRNA synthetase, an enzyme necessary for protein production, binds to the NAE1 subunit of E1 to capture and protect activated UBE2M before it reaches the downstream target [52].

**Fig. 2:**
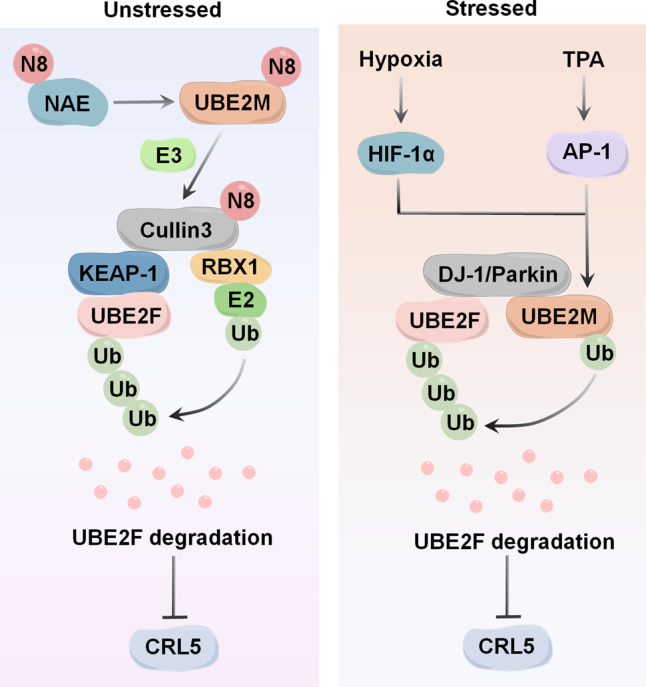
UBE2M acts as a dual E2 for neddylation and ubiquitylation to degrade UBE2F. Under physiological conditions, UBE2M serves as a neddylation E2 to promote CUL3 neddylation, triggering polyubiquitylation and degradation of UBE2F through CUL3-KEAP1 E3 ligase. Under stressed conditions, UBE2M is transcriptionally activated by HIF-1α or AP-1, which makes it serve as a ubiquitylation E2 to complex with DJ-1/Parkin to promote the ubiquitylation and degradation of UBE2F. Ultimately, the degradation of UBE2F is accompanied by the inactivation of CRL5. TPA: a typical tumor promoter and mitogen stimulator, inducing c-JUN.

The enzymes UBE2M and UBE2F can activate distinct cullins by enhancing neddylation modification, and interestingly, there is a cross-talk that leads to UBE2M targeting UBE2F for degradation [53]. Specifically, UBE2M serves as a stress-inducible protein and a dual E2 for neddylation and ubiquitylation to degrade UBE2F. Under physiological conditions, UBE2M acts as a neddylation E2 to promote CUL3 neddylation, which triggers polyubiquitylation and degradation of UBE2F through CUL3-KEAP1 E3 ligase. However, UBE2M is transcriptionally activated under stress by hypoxia-inducible factor 1α (HIF-1α) or transcription factor AP-1 (AP-1). UBE2M performs ubiquitylation E2 for Parkin-DJ-1 E3, followed by ubiquitylation and degradation of UBE2F. Ultimately, the degradation of UBE2F is accompanied by the inactivation of CRL5. Collectively, these findings provide evidence that one neddylation E2 (UBE2M) acts as a dual E2 for both neddylation and ubiquitylation to decrease the protein levels of the other (UBE2F), leading to one CRL E3 (CRL3) inactivating the other (CRL5) (Fig. 2).

## UBE2M and UBE2F as attractive anti-tumor targets

**Fig. 3:**
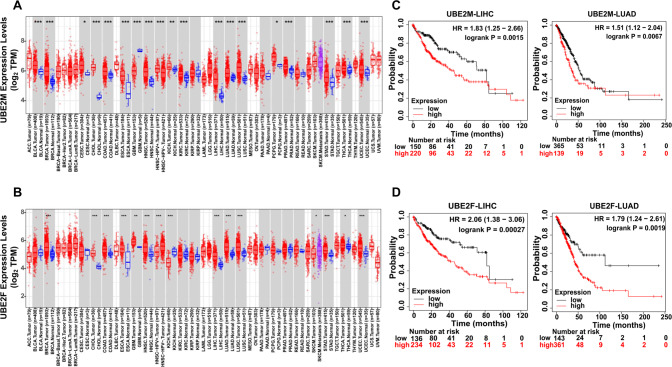
Expression of UBE2M and UBE2F in cancers and their effects on the prognosis of patients with LUAD and LIHC. **A**, **B** The expression of UBE2M and UBE2F in different types of cancer was investigated with the TIMER database. The variance was similar between the groups that were being compared. *P* < 0.05 was considered as statistical significance. *P < 0.05, **P < 0.01, ***P < 0.001 for the indicated comparison. **C**, **D** Kaplan-Meier analysis shows that increased levels of both UBE2M and UBE2F are related to worse survival for patients with lung adenocarcinoma (LUAD) or with liver hepatocellular carcinoma (LIHC).

**Fig. 4:**
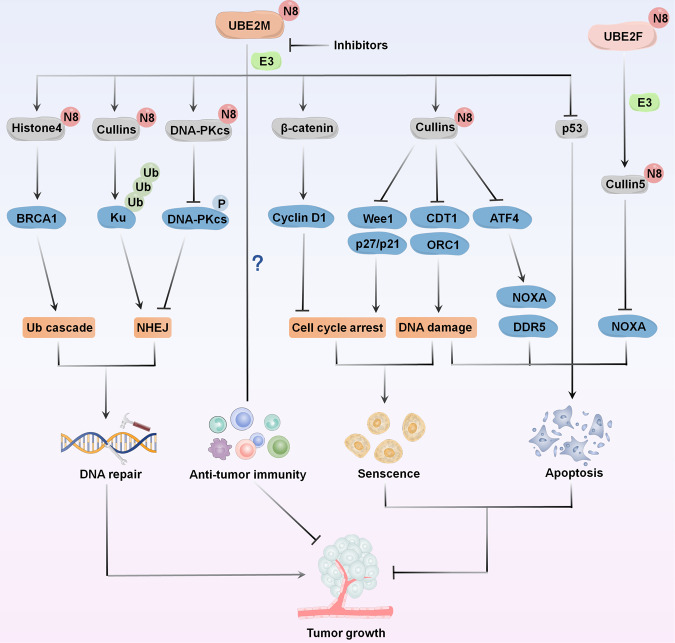
The function of UBE2M and UBE2F in tumor growth. UBE2M influences tumor growth by modulating several biological responses of tumor cells, such as DNA-damage response, cell cycle arrest, apoptosis, or senescence. UBE2F promotes the degradation of NOXA and then inhibits apoptosis and induces tumor growth.

Most studies reveal that both two neddylation E2s act as oncogenes, evidenced mainly by their significant upregulation in various human cancers, including esophageal squamous cell carcinoma, osteosarcoma, lung cancer, and hepatocellular carcinoma [54–59]. In addition, the upregulation of the two neddylation E2s correlates closely with illness progression [55–58]. To attain a greater understanding of the expression profile and prognostic significance of UBE2M and UBE2F in human cancers, we examined their expression with the aid of Tumor Immune Estimation Resource (TIMER) online database, an interactive platform that allows users to conduct in-depth studies of TCGA gene expression profiles (http://timer.cistrome.org/). Compared to normal tissues, the mRNA levels of UBE2M are elevated in 17 different kinds of human cancers, while the mRNA levels of UBE2F are elevated in 12 different types of human cancers (Fig. 3A, B). Kaplan–Meier analysis shows that increased mRNA levels of both enzymes correlated to worse survival for patients with lung adenocarcinoma (LUAD) or with liver hepatocellular carcinoma (LIHC) (Fig. 3C, D). They exert oncogenic effects by enhancing the neddylation of certain substrates to mediate a variety of signaling pathways and modulate multiple biological activities, such as apoptosis or senescence (Fig. 4). These findings suggest that the overactivation of these two enzymes could be an oncogenic event throughout the process of tumor occurrence and development.

## Targeting UBE2M for anti-tumor therapy

In recent decades, increasing studies have shown UBE2M, also known as UBC12, to be an attractive anti-tumor target. Compared with E1 subunits (NAE1 and UBA3), UBE2M is significantly more consistent with the global protein neddylation levels [56]. Targeting UBE2M suppresses the growth of MLN4924-resistant cells by inhibiting cullins neddylation and inducing the accumulation of CRLs substrates [56]. Moreover, UBE2M-mediated protein neddylation is essential for multiple cellular responses, such as DNA-damage response (DDR), apoptosis, senescence, and anti-tumor immunity. These findings validate UBE2M as an attractive alternative anti-tumor target to efficiently inhibit the neddylation pathway.

## UBE2M participates in DDR

DNA double-strand break (DSB), contributing significantly to genomic stability [60, 61], is sensed and repaired by DDR, warranting the recruitment and post-translational modification of multiple proteins at the damaged DNA sites. This phenomenon induces checkpoint signaling or essential repair steps [62, 63]. Recent studies have shown that UBE2M-mediated neddylation of cullins, or non-cullins, is involved in the DDR pathway (Fig. 4). UBE2M affects DDR and the integrity of the genome by regulating several CRLs substrates, such as CDT1, p21, and claspin, which play complex roles in the increased DNA-damage in UBE2M-silenced cells [64]. Another study supported the accumulation effects of CRLs substrates CDT1 and ORC1 caused by silenced UBE2M. This phenomenon subsequently induces DSBs, evidenced by the upregulated expression of γ-H2AX [65].

Generally, Ku70/Ku80 (Ku) heterodimer is first recruited to the DSB sites, after which, DNA-dependent protein kinase catalytic subunit (DNA-PKcs) is recruited into the process, facilitating nonhomologous end-joining (NHEJ) repair [66, 67]. Upon DNA-damage, UBE2M-mediated cullins neddylation promotes ubiquitylation of Ku, releasing it and its associated proteins from the damaged sites after repair. After NHEJ, ionizing radiation hypersensitivity and decreased cell survival occur in UBE2M-depleted cells [68]. Moreover, UBE2M also regulates the neddylation of DNA-PKcs, which promotes DNA-PKc autophosphorylation, preferentially activating the NHEJ pathway and facilitating its release from DNA-damage sites after Ku [69]. Consistently, the knockdown of UBE2M significantly enhances the sensitivity of hormone-resistant prostate cancer cells to radiation-induced DNA damage [70].

Furthermore, in response to various stimuli, NEDD8 may accumulate at DNA-damage sites by relying on UBE2M rather than UBE2F. UBE2M, together with an E3 ubiquitin ligase RNF111, promotes ionizing radiation-induced histone H4 neddylation and links another E3 ligase RNF168 to DNA-damage sites. This occurrence leads to the recruitment of BRCA1 and other downstream DDR factors to repair the damaged DNA [71]. Notably, another group has reported that UBE2M/RNF111-mediated neddylation inhibits BRCA1 and CtIP-regulated DNA end resection, an essential mechanism aiding the selection of an appropriate repair pathway [72].

## UBE2M inhibition induces cell cycle arrest

The aberrant functioning of cell cycle regulators leads to unregulated cell proliferation, making them promising therapeutic targets for cancer treatment [73, 74]. UBE2M has been shown to crucially regulate the tumor-suppressive cell cycle inhibitors (Fig. 4). In lung cancer and esophageal squamous cell carcinoma cells, UBE2M knockdown disturbs cell cycle progression by triggering G2 phase cell cycle arrest, specifically by inhibiting cullins neddylation and upregulating CRL substrates (p21, p27, and Wee1) [55, 56]. In hepatocellular carcinoma cells, UBE2M-mediated stabilization of β-catenin, leading to the upregulation of its downstream effectors, known as cyclin D1, promotes the G1/S transition of cells [75]. These data suggest that suppression of UBE2M can potentially trigger cell cycle arrest at distinct stages in a cell type-dependent manner via multiple mechanisms.

## UBE2M inhibition induces apoptosis or senescence

UBE2M plays an essential role in regulating apoptosis and senescence (Fig. 4). In intrahepatic cholangiocarcinoma cells, UBE2M knockdown induces apoptosis, demonstrated by shrinkage of cellular morphology and the upregulation of cleaved PARP and caspase-3/-9 [65]. In hepatocellular cells, UBE2M knockdown promotes apoptosis by inducing the accumulation of cleaved PARP and caspase-3 and increasing the mRNA levels of apoptosis-associated proteins, including p53, PUMA, and Bax [76]. Interestingly, UBE2M knockdown leads to p53-mediated apoptosis by activating and stabilizing the protein [76]. In esophageal squamous cell carcinoma cells, UBE2M knockdown triggers apoptosis or senescence in a cell line-dependent manner. EC1 cells with UBE2M knockdown exhibit typical senescent morphology, well-characterized by an enlarged and flattened cellular shape and positive staining for senescence-related β-galactosidase [55]. In contrast, KYSE450 cells with UBE2M knockdown show prominent apoptotic features, shrunk morphology, and a substantial increase in cell numbers positive for annexin V [55]. Moreover, UBE2M knockdown increases the accumulation of CRL substrate activating transcription factor 4 (ATF4), activating death receptor 5 (DR5)-mediated extrinsic apoptosis and proapoptotic protein NOXA-mediated intrinsic apoptosis [55, 77, 78]. Thus, induction of apoptosis or senescence establishes UBE2M as a promising anti-tumor target.

## UBE2M is involved in anti-tumor immunity

**Fig. 5:**
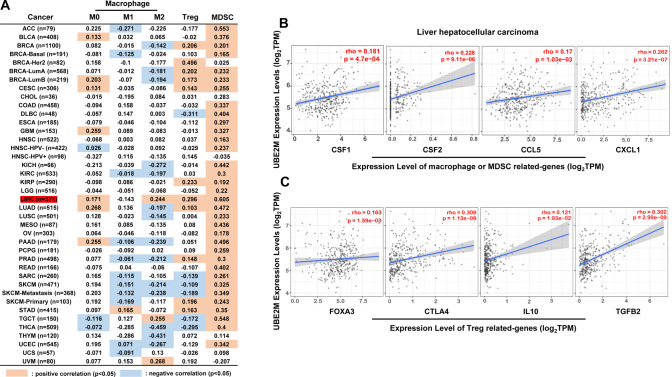
The correlation of UBE2M and immune suppressive cell populations. **A** Graphs generated from TIMER database show the correlations between UBE2M and the abundance of macrophage, Treg, and MDSCs. **B**, **C** The expression of UBE2M was significantly correlated with multiple genes that are related to macrophage M2, MDSCs, or Treg in liver hepatocellular carcinoma. Spearman correlation analysis was used to assess the correlation. *P* < 0.05 was considered as statistical significance.

Immune evasion is a hallmark of cancer. Anti-tumorigenic immune cells are often exhausted or repressed by immune suppressive cell populations, such as tumor-associated macrophages (TAMs), regulatory T (Treg), and myeloid-derived suppressor cells (MDSCs), leading to tumor immune evasion [79–81]. Hence, targeting these immune suppressive cells is a promising anti-tumor immunotherapy. It is known that UBE2M knockdown inhibits the expression of proinflammatory cytokines triggered by exposure to lipopolysaccharides (LPS) (e.g., IL-6 and TNF-α) in macrophages. UBE2M knockdown suppresses CUL1 neddylation, inactivates CRL1, and induces the accumulation of phosphorylated IκBα and the subsequent suppression of NF-κB nuclear translocation. This phenomenon transcriptionally inhibits macrophage-associated cytokines [82]. Recent reports suggest that the Ube2m/Rbx1 axis, rather than the Ube2f/Rbx2 one, is crucial to the homeostasis and survival of Treg cells [83]. We then investigated the association between UBE2M expression and the abundance of immune cell populations in tumors using the TIMER database to assess further the effects of UBE2M on these immune suppressive cells (Fig. 5A). This analysis revealed that in hepatocellular carcinoma, the expression of UBE2M was significantly positively correlated with the abundance of Treg, MDSCs, and macrophage M2 (Fig. 5A). Moreover, the expression of UBE2M was also significantly correlated with multiple genes related to macrophage M2, MDSCs, or Treg (Fig. 5B, C). These results collectively suggest that UBE2M may drive immunosuppression in hepatocellular carcinoma. However, future investigation of detailed mechanisms is warranted.

Targeting immune checkpoints mediated by programmed cell death 1 (PD-1) and its ligand PD-L1 is a practical approach to enhance anti-tumor immunity. This line of therapy has been approved for treating various human cancers with durable clinical benefits [84, 85]. Notably, inhibition of UBE2M-mediated neddylation significantly upregulates the expression of PD-L1 by inactivating CUL1 and CUL3 in glioblastoma cancer cells. This phenomenon is mainly achieved by the transcriptional activation of PD-L1 by dysregulating the CUL1-FBXW7/c-MYC axis and stabilizing PD-L1 protein by inhibiting CUL3 E3 ligase activity, leading to T-cell exhaustion [86]. Moreover, inhibition of CUL3-SPOP E3 ligase impairs ubiquitination-mediated PD-L1 degradation, increasing PD-L1 protein levels and reducing the numbers of tumor-infiltrating lymphocytes in mouse tumors and primary human prostate cancer [87]. A recent study also revealed that inhibition of the neddylation pathway by MLN4924 activates ERK and JNK signals, leading to AP-1 activation. Activated AP-1 transactivates PD-L1 expression, inducing tumor immune evasion to fight the anti-tumor activity of MLN4924 [88]. These findings offer novel insights for further clinical experiments on tumor patients using a combined method of UBE2M targeting and anti-PD-L1/PD-1 therapy. Altogether, the identification of the role of UBE2M in modulating anti-tumor immunity deserves further research.

## Targeting UBE2M-DCN1 interaction for regulation of neddylation pathway

Efforts have been directed to develop more specific small-molecule inhibitors that preferentially target neddylation E2s to address the shortcomings of MLN4924. The co-crystal structure assessment of DCN1, which has no RING finger domain and acts as a co-NEDD8-E3 with RBX1, and its binding partners UBE2M, harbors the potential to develop strong small-molecule inhibitors [47, 51, 89–91]. Over the past few years, several different research facilities have developed small-molecule inhibitors for suppressing interactions between UBE2M and DCN1. Zhou et al. created potent peptidomimetics, such as DI-591 and DI-404, by significantly modifying the N-terminal 12-residue peptide of UBE2M to inhibit UBE2M-DCN1 interaction [92, 93]. Subsequently, Guy et al. identified non-peptidic and potent small-molecule UBE2M-DCN1 inhibitors, such as NAcM-HIT, by high-throughput screening of over 600,000 compounds. The chemical optimization of NAcM-HIT led to designing two more potent inhibitors, NAcM-OPT and NAcM-COV [94–96]. Liu et al. discovered the triazolo[1,5-α]pyrimidine-based inhibitor WS-383, which targets the UBE2M-DCN1 interaction [97]. In addition, Zhao et al. discovered small-molecular DCN1 inhibitors DC-1 and DC-2 based on pyrimidines [98].

Such studies have successfully discovered potent small-molecule inhibitors that are either covalent or non-covalent with powerful affinities to DCN1. These UBE2M-DCN1 inhibitors have been shown to efficiently block the neddylation of CUL1 and/or CUL3 while exerting no or minimal impact on the other members of the cullin family. These UBE2M-DCN1 inhibitors, as predicted, cause an accumulation of the CUL1 or CUL3 substrates, such as p21, p27, and NRF2 [92, 95–98]. However, compared to MLN4924, the UBE2M-DCN1 inhibitors have moderate cellular potency, suggesting the need for further potency improvement.

## Targeting UBE2F for anti-tumor therapy

In contrast with many studies done on UBE2M, UBE2F receives little attention. Recently, UBE2F has been shown to inhibit apoptosis and induce cell growth. UBE2F can be efficiently targeted as a chemo-/radiosensitizing strategy by triggering apoptosis.

## UBE2F inhibition induces apoptosis

Zhou et al. reported that by coupling to RBX2, UBE2F neddylates CUL5, which activates CRL5, eventually leading to the ubiquitylation and degradation of NOXA through the K11-linkage [57]. In lung cancer cells, overexpression of UBE2F activates CRL5 and promotes NOXA degradation, leading to inhibition of apoptosis and improvement of cell survival [57]. HA-9104 is recently discovered as a novel small-molecule inhibitor targeting UBE2F-CRL5 axis. HA-9104 interacts with UBE2F to reduce its protein levels (via a yet-to-defined mechanism), thereby inhibiting CUL5 neddylation. Blockage of CUL5 neddylation results in CRL5 inhibition and NOXA accumulation to trigger apoptosis [48]. Since UBE2F inhibition promotes apoptosis and suppresses cancer cell growth (Fig. 4), it is a promising target for anti-tumor therapy.

Moreover, peroxiredoxin PRDX1 binds to UBE2F and CUL5 to form a triple-molecule complex, PRDX1-UBE2F-CUL5, essential for CUL5 neddylation. Silencing PRDX1 or blocking PRDX1 oligomerization significantly inhibits CUL5 neddylation, suppressing NOXA ubiquitination and degradation. Etoposide, an anti-cancer chemotherapeutic DNA damaging agent, increases NOXA transcription, leading to apoptosis. Colorectal cancer cells increase CUL5 neddylation to accelerate NOXA degradation, which prevents etoposide-induced apoptosis. At the same time, the knockdown of PRDX1 eliminates etoposide-induced CUL5 neddylation and increases the sensitivity of colorectal cancer cells to etoposide therapy [99].

## UBE2F serves as a target for chemo-/radiosensitization

UBE2F, an apoptotic regulatory protein, is a probable viable target for chemosensitization [100, 101]. In lung cancer cells, depletion of UBE2F renders the cells more sensitive to multiple anti-tumor agents (e.g., an inhibitor of anti-apoptotic protein MCL1) by accumulating NOXA [101]. UBE2F upregulation allows lung cancer cells to evade apoptosis caused by platinum exposure. Mechanistically, platinum prevents the generation of the complex required for proteasome-mediated UBE2F degradation, which ultimately results in UBE2F accumulation. This phenomenon demonstrates an increase in the CUL5 neddylation level, consistent with the decreased protein levels of NOXA. UBE2F knockdown dramatically increases cell sensitivity to platinum therapy by increasing NOXA protein levels and consequently promoting apoptosis [100]. Notably, these effects manifest in other cancer cells, such as breast and ovarian cancer cells, indicating the role of UBE2F, a universal drug target of platinum-sensitization [100]. Nevertheless, the expressions of other neddylation factors, such as UBE2M, are not affected following platinum treatment [100].

Besides chemosensitization, targeting UBE2F also displays a sensitizing effect on radiotherapy [102]. Elevated UBE2F levels can be attributed to oxidative stress induced by irradiation or other stimuli, causing the degradation of ROS-induced NOXA, consequently inducing apoptotic resistance to radiotherapy. Moreover, silencing UBE2F suppresses NOXA degradation and increases cancer cells’ susceptibility to irradiation-mediated apoptosis [102]. Taken together, UBE2F-mediated activation of CRL5 and subsequent ubiquitylation and degradation of NOXA potentially hold great promise as both an anti-tumor target and a chemo-/radiosensitizing target.

## Conclusions

Targeting the overactivated neddylation pathway has been demonstrated as a promising anti-tumor strategy, supported by the development of MLN4924, a potent inhibitor of the neddylation E1 subunit UBA3. However, the emergence of drug-resistant mutations in UBA3 warrants the identification of alternative targets against the neddylation pathway. Recent and ongoing research has revealed that UBE2M and UBE2F perform an integral function in the biology of tumors. Overexpression of both UBE2M and UBE2F in cancer cells is associated with increased cell proliferation and poor survival. UBE2M regulates tumor growth by modulating several cellular responses, such as DDR, senescence, or apoptosis. The functional role of UBE2F, on the other hand, remains poorly characterized. Recent studies have shown that UBE2F inhibits apoptosis, induces cell growth, and serves as an effective chemo-/radiosensitizing target. However, the effects of both E2s on anti-tumor immunity undoubtedly demand more experimental investigations. In-depth elucidation of the mechanisms of these E2s may provide more in-depth knowledge for targeting UBE2M and UBE2F as attractive anti-tumor therapy.

Many inhibitors targeting UBE2M-DCN1 interaction have been discovered to overcome the limitations of MLN4924 and tackle the crucial role of UBE2M on the neddylation pathway and tumor growth. However, compared to MLN4924, the tumor cell-killing potency of these UBE2M-DCN1 inhibitors is moderate, suggesting the need for further potency improvement. Promoting anti-tumor clinical trials of these E2 inhibitors alone or in combination may direct further research. Recent studies reported that, in addition to effective tumor treatment, MLN4924 also plays a potential role in the treatment of obesity [103], insulin resistance [103, 104], nonalcoholic fatty liver [105], and ischemia-reperfusion injury [106–108]. Hence, it would be interesting to identify the function of these UBE2M-DCN1 inhibitors in these non-tumor diseases.

In summary, the current research offers profound insights into the role of UBE2M and UBE2F in tumor progression, which is highly conducive to aiding the development of targeted inhibitors with higher potency and selectivity.

## Data Availability

The corresponding author will provide the data and materials upon reasonable request.
